# Regulatory T Cell–Derived TRAIL Is Not Required for Peripheral Tolerance

**DOI:** 10.4049/immunohorizons.2000098

**Published:** 2021-01-22

**Authors:** Rebekah E. Dadey, Stephanie Grebinoski, Qianxia Zhang, Erin A. Brunazzi, Amanda Burton, Creg J. Workman, Dario A. A. Vignali

**Affiliations:** * Department of Immunology, University of Pittsburgh School of Medicine, Pittsburgh, PA 15261; † Tumor Microenvironment Center, University of Pittsburgh Medical Center Hillman Cancer Center, Pittsburgh, PA 15232; ‡ Graduate Program of Microbiology and Immunology, University of Pittsburgh School of Medicine, Pittsburgh, PA 15213; § Department of Immunology, St. Jude Children’s Research Hospital, Memphis, TN 38105; ¶ Cancer Immunology and Immunotherapy Program, University of Pittsburgh Medical Center Hillman Cancer Center, Pittsburgh, PA 15232

## Abstract

TRAIL (*Tnfsf10*/TRAIL/CD253/Apo2L) is an important immune molecule that mediates apoptosis. TRAIL can play key roles in regulating cell death in the tumor and autoimmune microenvironments. However, dissecting TRAIL function remains difficult because of the lack of optimal models. We have now generated a conditional knockout (*Tnfsf10*^L/L^) for cell type–specific analysis of TRAIL function on C57BL/6, BALB/c, and NOD backgrounds. Previous studies have suggested a role for TRAIL in regulatory T cell (T_reg_)–mediated suppression. We generated mice with a T_reg_-restricted *Tnfsf10* deletion and surprisingly found no impact on tumor growth in C57BL/6 and BALB/c tumor models. Furthermore, we found no difference in the suppressive capacity of *Tnfsf10*-deficient T_regs_ and no change in function or proliferation of T cells in tumors. We also assessed the role of TRAIL on T_regs_ in two autoimmune mouse models: the NOD mouse model of autoimmune diabetes and the myelin oligodendrocyte glycoprotein (MOG) C57BL/6 model of experimental autoimmune encephalomyelitis. We found that deletion of *Tnfsf10* on T_regs_ had no effect on disease progression in either model. We conclude that T_regs_ do not appear to be dependent on TRAIL exclusively as a mechanism of suppression in both the tumor and autoimmune microenvironments, although it remains possible that TRAIL may contribute in combination with other mechanisms and/or in different disease settings. Our *Tnfsf10* conditional knockout mouse should prove to be a useful tool for the dissection of TRAIL function on different cell populations in multiple mouse models of human disease.

## INTRODUCTION

TRAIL (*Tnfsf10*/CD253/Apo2L) is a homotrimeric type II transmembrane TNF superfamily member (1–3). TRAIL, discovered because of its similarity to Fas, is a molecule that induces extrinsic apoptosis (4, 5). This signal is delivered through TRAIL binding to agonistic murine death receptor (DR)5 (*Tnfrsf10b*) or human DR4/TRAILR1 (*Tnfrsf10a*) and DR5/TRAILR2 (*Tnfrsf10b*) (6, 7). Receptor–ligand interaction recruits adaptor molecule FADD, which recruits and activates initiator caspases such as caspase 8 and 10 (8, 9). The initiators will then cleave and activate an executioner caspase, such as caspase 3, which will degrade cellular components, ultimately leading to cellular apoptosis (10). TRAIL can induce cell death as either a membrane bound or soluble mediator, as it can be cleaved by intracellular aspartic and/or cysteine proteases (7, 11, 12). TRAIL expression is regulated by cell stimulation and presence of type 1 and type 2 IFNs on multiple cell types including T cells, NK cells, monocytes, macrophages, and dendritic cells (13–22). This upregulation can enable TRAIL-expressing cells to cytotoxically target ligand-expressing cells in various environments.

TRAIL was initially discovered as a molecule that specifically targets malignant cells and spares nonmalignant cells. TRAIL- or DR5-deficient mice are more susceptible to tumor growth and metastasis, implicating an important role for TRAIL in controlling tumor growth (23–31). This tumor-specific killing is primarily mediated by NK cells and CD8^+^ T cells in the tumor microenvironment (TME), although other cells express TRAIL in the TME (17, 31, 32). Moreover, although TRAIL is a molecule that targets cell death, it can also regulate immune cell function and proliferation (33).

Regulatory T cells (T_regs_) are an immunosuppressive subset of CD4^+^ T cells that can suppress activated immune cells and limit autoimmunity. For example, T_regs_ are critical for limiting multiple models of autoimmunity such as the NOD mouse, a spontaneous model of autoimmune diabetes, and the myelin oligodendrocyte glycoprotein (MOG) C57BL/6 model of experimental autoimmune encephalomyelitis (EAE). T_reg_ depletion in these models rapidly results in overt diabetes and exacerbated EAE disease severity, respectively (34–36). Despite this important role, T_regs_ can also suppress the antitumor response and therefore are an effective barrier to limiting tumor growth (37, 38). T_regs_ have multiple mechanisms of suppression and can use these mechanisms in the TME and autoimmune environment. T_regs_ can suppress through production of inhibitory cytokines, targeting of dendritic cell function, metabolic disruption, and direct cytolysis (39–41). Our laboratory has shown that T_regs_ from IL-10– and IL-35–deficient C57BL/6 mice upregulated TRAIL to suppress responding T cells and that T_regs_ from BALB/c mice express higher levels of TRAIL than T_regs_ from C57BL/6 mice (42). In addition, it has been reported that T_regs_ produce TRAIL in an allogenic skin graft model to suppress activated T cells (43). Taken together, these observations suggest that T_regs_ can use TRAIL to suppress immune cells in various disease environments.

In this study, we had two specific goals: 1) investigate TRAIL function in an inducible, cell type–specific manner by generating *Tnfsf10*^L/L^ mice on C57BL/6, BALB/c, and NOD backgrounds, as studies thus far have only used blocking Abs or constitutive *Tnfsf10* knockout mice, and 2) assess if T_regs_ require and/or are dependent on TRAIL as a mechanism of suppression within the tumor or autoimmune microenvironment by use of *Tnfsf10*^L/L^*Foxp3*^Cre^ mice.

## MATERIALS AND METHODS

### Mice

*Foxp3*^Cre-YFP^ mice on a C57BL/6 background were obtained from A. Y. Rudensky (Memorial Sloan-Kettering) (44). *Foxp3*^Cre^ mice on a BALB/c background were obtained from S. Sakaguchi (Osaka University) (45). Foxp3^Cre-GFP^.NOD mice were obtained from J. A. Bluestone (University of California, San Francisco) (46). All animal experiments were performed in the American Association for the Accreditation of Laboratory Animal Care–accredited, specific pathogen-free facilities in Division of Laboratory Animal Resources, University of Pittsburgh School of Medicine. Female and male mice of 4–6 wk of age were used for B6 and BALB/c experiments. All tumor phenotype and functional experiments were performed at 12 d after tumor inoculation unless otherwise specified. Female and male NOD mice were followed for diabetes incidence up to 30 wk of age. All NOD phenotype and functional experiments were performed with female mice at 10 wk unless otherwise specified. Animal protocols were approved by the Institutional Animal Care and Use of Committees of University of Pittsburgh.

### Generation of a Tnfsf10^L/L^ mouse

The *Tnfsf10*^L/L^ targeting construct was generated using standard recombineering methods (47). Initially, 26.7 kb of the *Tnfsf10* locus were retrieved from a bacterial artificial chromosome plasmid and an Loxp-Neo-Loxp cassette inserted 313 bp upstream of exon 2. The Neo was removed via Cre-mediated recombination, leaving a single Loxp and an *Stu*I restriction site (inserted into the intron of the retrieved *Tnfsf10* locus). An Frt-Neo-Frt-Loxp cassette was then inserted 573 bp downstream of exon 5 to establish an alter-nate exon 2 containing the following: a *Spe*I restriction site, the splice acceptor from exon 2, “self-cleaving” T2A peptide sequence, a truncated version (nonfunctioning) of the human nerve growth factor receptor (hNGFR), and the SV40 polyadenylation sequence. The linearized targeting construct was electroporated into JM8A3.N1 embryonic stem cells (C57BL/6N background) and neomycin-resistant clones were screened by Southern blot analysis using *Stu*I and *Spe*I restriction digests for the 5′ and 3′ ends, respectively. Correctly targeted clones were 100% normal diploid by karyotype analysis and were injected into C57BL/6 blastocysts. Chimeric mice were mated to C57BL/6 mice and transmission of the targeted allele verified by PCR. The mice were crossed with actin flippase mice to remove the Neo cassette. The mice were backcrossed >10 generations onto the BALB/c or NOD background and verified by microsatellite analysis. Genotyping primers are 5′-GCCCACGGGTGTAAAGAGCAGTTC-3′, 5′-GGTGGAACAGCTGACAGACATGATAAGATAC-3′, and 5′-GTCTCCCCAGTCCAATCACTGCTAC-3′. Primers for detection of exon 1 of *Tnfsf10* are forward 5′-GCACTCCGCCTTCTAACTGT-3′ and reverse 5′-GTGCTGACTGAAGCTGAGGT-3′, exon 2 forward 5′-GACGGATGAGGATTTCTGGGAC-3′ and reverse 5′-TTCAATGAGCTGATACAGTTGCC-3′, and exon 5 forward 5′-ATGGAAAGACCTTAGGCCAGA-3′ and reverse 5′-TAGATGTAATACAGGCCCTCCTGC-3′.

### Measurement of diabetes and insulitis

Measurement of diabetes and insulitis were performed as previously described (48–50). Briefly, diabetes incidence was monitored weekly through presence of glucose in the urine with Diastix (Bayer). Mice positive for glucose on Diastix were then measured for blood glucose with a Breeze2 glucometer (Bayer). Mice were considered diabetic and were marked for sacrifice when blood glucose was ≥400 mg/dl.

Pancreata for histology were prepared as previously described at the University of Pittsburgh Biospecimen Core (48). Briefly, pancreata were embedded in a paraffin block and cut into 4-μm-thick sections with 150-μm steps between sections and stained with H&E. An average of 60–80 islets per mouse were scored in a blinded manner. Two methods of insulitis measurement were used as previously described (51).

### Islet isolation and lymphocyte preparation

Islets were prepared as previously described (48, 52). Briefly, 3 ml of collagenase (600 U/ml in complete HBSS with 10% FBS) was perfused through the pancreatic duct. Pancreata were then incubated for 30 min at 37°C. Pancreata were then washed two times and resuspended in clear complete HBSS with 10% FBS, and islets were isolated by hand under a dissecting microscope. Isolated islets were dissociated with 1 ml dissociation buffer (Life Technologies) for 15 min at 37°C with vortexing every 5 min. Cells were washed, resuspended, counted, and used.

### EAE induction

Induction of EAE was performed as described previously (53, 54). Briefly, IFA (Difco) at was supplemented with 5 mg/ml *Mycobacterium tuberculosis* (Difco) to make CFA. MOG peptide (AAPPTec) was diluted to 1 mg/ml in PBS, and the CFA and MOG peptide were mixed at a 1:1 ratio. Mice were injected with 100 μl of the emulsion on both flanks s.c. Pertussis toxin (200 ng/200 μl PBS; Sigma-Aldrich) was injected i.p. on day 0 and day 2 of injection. Animals were scored blinded for clinical symptoms as follows: 0, no change; 1, limp tail; 2, partial hind limb paralysis; 3, full hind limb paralysis; 4, full hind limb paralysis and partial front limb paralysis; and 5, moribund or death.

### Cell staining, flow cytometry, and purification

Single-cell suspensions were stained with Abs for CD4 (GK1.5; BioLegend), CD8a (53–6.7; BioLegend), TCRβ (H57–597; eBioscience), cleaved caspase (Asp175; Cell Signaling Technologies, CST), CD45.2 (104; BioLegend), Foxp3 (FJK-16s; eBioscience), Ki67 (B56; BD Biosciences), TNF-α (MP6-XT22; BioLegend), IFN-γ (XMG1.2; BioLegend), DR5 (MD5–1; BioLegend), LAP-TGF-β (TW7–16B4; BioLegend), IL-10 (JES5–16E3; BioLegend), CTLA-4 (UC10–4B9; BioLegend), CD73 (TY/11.8; BioLegend), CD39 (24DM51; BioLegend), CD11c (N418; BioLegend), CD19 (ID3; BD Biosciences), F4/80 (BM8; BioLegend), NK1.1 (PK136; eBioscience), CD49b (DX5; BioLegend), and insulin (182410; R&D Systems). Surface staining was performed on ice for 15 min. Dead cells were discriminated by staining with Ghost Viability Dye (Tonbo Biosciences) in PBS prior to surface staining. For cytokine expression analysis, cells were activated with 100 ng/ml PMA (Sigma-Aldrich) and 500 ng/ml ionomycin (Sigma-Aldrich) in complete RPMI 1640 containing 10% FBS and monensin (eBioscience) for 4 h. For intracellular staining of cytokines and transcription factors, cells were stained with surface markers, fixed in Fix/Perm buffer (eBioscience) for 45 min, washed twice in permeabilization buffer (eBioscience), and stained in permeabilization buffer for 30 min on ice. Immunostaining for Ki67 was performed using the BD Cytofix/Cytoperm kit. Samples were acquired on a Fortessa (BD Biosciences) and analyzed by FlowJo (Tree Star) or sorted on an Aria II (BD Biosciences). Identification of various immune cell populations was first subgated on live CD45.2^+^ cells. From this gate, the following strategy for each population was used: TCRβ^+^CD4^+^Foxp3^−^ (in this study referred to as CD4^+^), TCRβ^+^CD4^+^Foxp3^+^ (T_reg_), TCRβ^+^CD8^+^ (CD8^+^), TCRβ^−^CD49b^+^ or TCRβ^−^ NK1.1^+^ (NK^+^), TCRβ^−^ CD11c^+^ (CD11c^+^), TCRβ^−^ F4/80^+^ (F4/80^+^), and all other TCRβ^−^ cells. Gating for sorting these populations remains the same except for the CD4^+^ Foxp3^−^ and T_reg_ populations. CD4^+^ Foxp3^−^ and T_reg_ populations used the following strategy, respectively: TCRβ^+^CD4^+^Foxp3(YFP)^−^ (C57BL/6) or TCRβ^+^CD4^+^CD25^−^(BALB/c) (CD4) and TCRβ^+^CD4^+^Foxp3(YFP)^+^ (C57BL/6) or TCRβ^+^CD4^+^CD25^+^ CD127^−^ (BALB/c) (T_reg_). NOD T_regs_ were isolated as TCRβ^+^CD4^+^Foxp3(GFP)^+^, and CD4s were isolated as TCRβ^+^CD4^+^Foxp3 (GFP)^−^.

### Tumor models

The B16.F10 were obtained from M. J. Turk (Dartmouth College) (55). The MC38 colon adenocarcinoma cells were obtained from J. P. Allison (MD Anderson Cancer Center) (56). The CT26 cells were obtained from R. Binder (University of Pittsburgh) (57). These cells were cultured as previously described (58). C57BL/6 mice were injected with 1.25 × 10^5^ B16 melanoma cells (intradermally [i.d.]) or 5.0 × 10^5^ MC38 colon carcinoma cells (s.c.). We treated mice injected with MC38 with isotype (Rat IgG2a; Leinco) or anti–programmed cell death (PD)-1 (Leinco) as previously described (59). Tumors were measured every 3 d with a digital caliper in two dimensions (width and length) and presented as tumor size (square millimeters; defined as *w* × *l*). BALB/c were injected with 1.25 × 10^5^ CT26 colorectal carcinoma s.c. and measured every 3 d for tumor growth. Tumors were prepared for single-cell suspension with an enzymatic digestion of collagenase IV (200 U/ml) and dispase (1 U/ml) in complete RPMI 1640 and mechanical disruption.

### In vitro assays

Microsuppression assays were performed as previously described (59, 60). Briefly, T_reg_ cells were isolated from the spleen of naive mice or nondraining lymph node (NDLN) and tumor-infiltrating lymphocytes (TIL) of mice 12 or 18 d after injection with B16 or CT26. Isolated T_regs_ were cocultured with CellTrace Violet (Life Technologies)–labeled CD4^+^Foxp3^−^ responder T cells in the presence of mitomycin C–treated, TCRβ-depleted splenocytes and anti-CD3ε (1 μg/ml) for 72 h at 37°C.

### mRNA isolation, cDNA synthesis, and quantitative PCR

Cell populations were isolated from naive *Foxp3*^Cre-YFP^.B6 or *Foxp3*^Cre^.BALB/c mice or from the NDLN and TIL of B16-bearing *Foxp3*^Cre-YFP^.B6 and *Tnfsf10*^L/L^ *Foxp3*^Cre-YFP^.B6 mice. Cells were isolated from NDLN, pancreatic draining lymph node, and islet from 10-wk-old female Foxp3^Cre-GFP^.NOD. RNA was extracted using the RNAeasy Micro Kit (QIAGEN). cDNA was produced using the High-Capacity cDNA Reverse Transcription Kit (Thermo Fisher Scientific) following the manufacturer’s instructions. EvaGreen-based quantitative PCR (qPCR) was performed using the following primers: *Tnfsf10* forward, 5′-TCTGTGGCTGTGACTTACATG-3′ and reverse, 5′-AAGCAGGGTCTGTTCAAGATC-3′; and *HPRT* forward, 5′-TCAGTCAACGGGGGACATAAA-3′ and reverse, 5′-GGGGCTGTACTGCTTAACCAG-3′. Relative quantification was determined via the δ CT method.

### Quantification and statistical analysis

Statistical analysis was performed with Prism version 8.0.0. Student *t* tests were used when only two experimental groups were involved. Tumor growth and EAE curves were analyzed using two-way ANOVA with multiple comparisons correction with sequential time point measurements. The log-rank (Mantel–Cox) test was used for diabetes incidence statistical analysis. Number of mice used in the experiment is represented by “*n*,” with number of individual experiments listed in legend. All *p* values were two sided, and statistical significance assessed at ≤0.05.

## RESULTS

### TRAIL is expressed on T_regs_ in the TME

**FIGURE 1.**
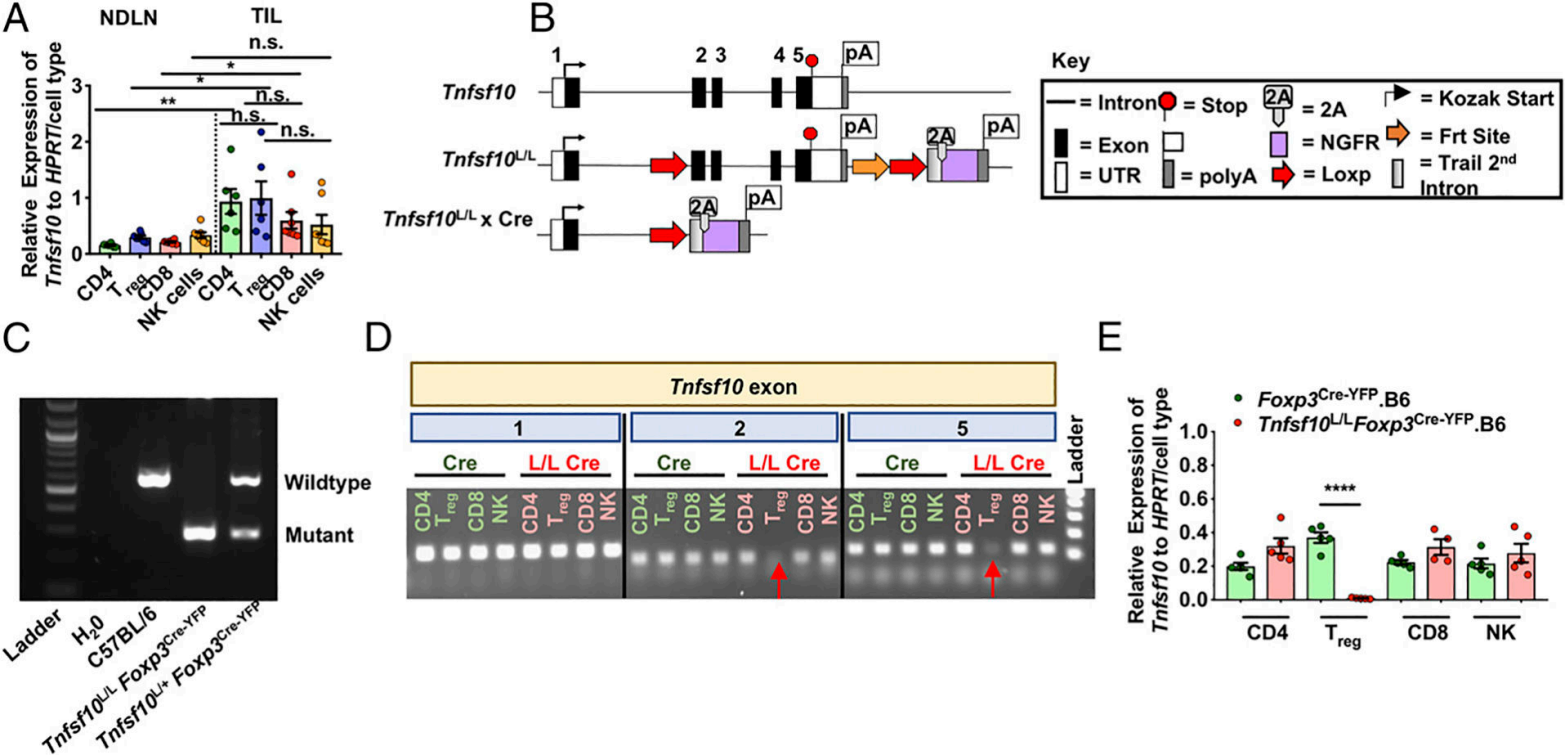
TRAIL is expressed on T_regs_ in the TME and generation of a *Tnfsf10*^*L/L*^ mouse. (**A**) C57BL/6 *Foxp3*^Cre-YFP^ mice were injected with 125,000 B16 cells i.d. and sacrificed 12 d postinoculation. Cells were sorted, and qPCR was performed for *Tnfsf10* and *HPRT*. (**B**) Schematic of the *Tnfsf10*^L/L^ mouse. (**C**) Genotyping PCR of genomic tail DNA of *Tnfsf10*^L/L^-targeted mice. (**D**) Cells were sorted from *Foxp3*^Cre-YFP^.B6 and *Tnfsf10*^L/L^ *Foxp3*^Cre-YFP^.B6 mice, genomic DNA isolated, and PCR performed using primers specific for exons 1, 2, and 5 of *Tnfsf10*. (**E**) Cells were sorted from *Foxp3*^Cre-YFP^.B6 and *Tnfsf10*^L/L^ *Foxp3*^Cre-YFP^.B6 mice and qPCR performed for *Tnfsf10* and *HPRT.* Data in (A) are representative of one experiment (*n* = 4–5 mice per group). Data in (C) and (D) are representative of one experiment (*n* = 1 mouse per group). (E) is representative of two experiments (*n* = 1–5 mice per group). Statistical analysis was determined by Student unpaired *t* test. *p < 0.05, **p < 0.01, ****p < 0.0001. ns, not significant.

We hypothesized that T_regs_ use TRAIL to suppress the antitumor response. Therefore, we initially assessed TRAIL expression in multiple cell populations isolated from the TME of B16 tumor–bearing mice, and we found substantial upregulation of *Tnfsf10* transcript in the TIL compared with the NDLN (Fig. 1A). Interestingly, T_regs_ and CD4^+^Foxp3^−^ were trending to have higher *Tnfsf10* levels in the TME compared with other cells in the TME. It is important to note that TRAIL protein expression was difficult to discern, as previously reported, which may be due to its low level of expression (61).

### Generation of a Tnfsf10^L/L^ mouse

To directly access the importance of TRAIL expression in distinct cell types in the TME, in particular in T_regs_, we generated a novel *Tnfsf10*^L/L^ mouse. LoxP sites were inserted in the intron between exons 1 and 2 and following exon 5 along with an artificial exon containing a truncated nonfunctional version of the hNGFR (Fig. 1B, 1C). The hNGFR was intended to serve as a reporter for Cre-mediated deletion of *Tnfsf10.* However, upon validation of the strain, it was found that expression of hNGFR was minimal following Cre-mediated deletion, likely because of the weak transcription strength of the *Tnfsf10* promoter consistent with challenges experienced in detected TRAIL expression (data not shown). This may also have been due to inefficient splicing into the artificial exon. To assess the role of TRAIL in T_regs_, we crossed the *Tnfsf10*^L/L^ mice with *Foxp3*^Cre-YFP^.B6 mice, and fidelity of T_reg_-specific deletion was verified by cell specific genotyping (Fig. 1D, 1E). Taken together, we have successfully generated a *Tnfsf10*^L/L^ murine model, thus enabling us to specifically examine the role of TRAIL in T_regs_.

### T_reg_-restricted deletion of Tnfsf10 does not affect tumor growth or suppression in C57BL/6 mice

**FIGURE 2.**
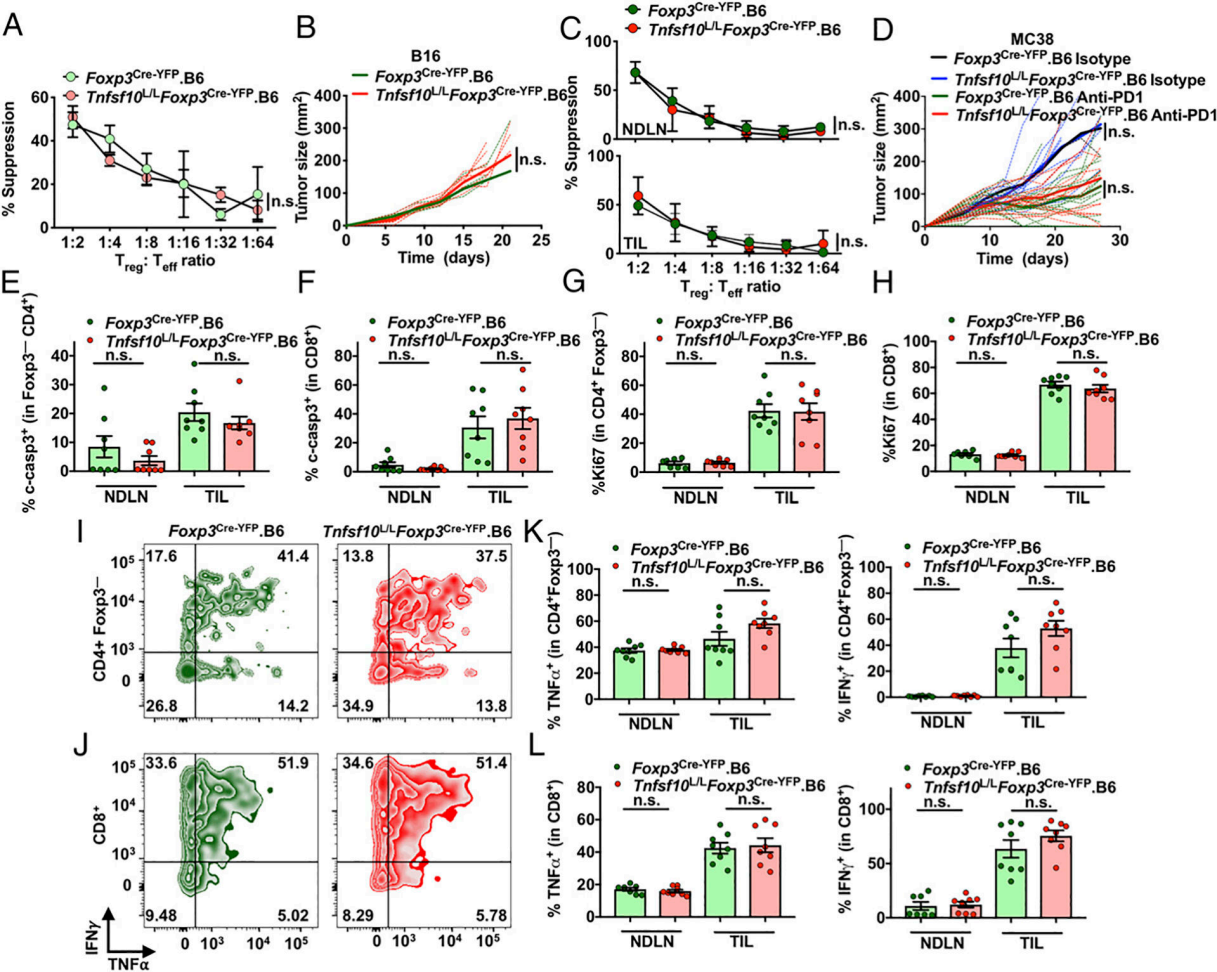
T_reg_-restricted deletion of *Tnfsf10* does not affect tumor growth or suppression in C57BL/6 mice. (**A**) T_regs_ were isolated from *Foxp3*^Cre-YFP^.B6 and *Tnfsf10*^L/L^ *Foxp3*^Cre-YFP^.B6 naive mice and cultured with effector CD4^+^ T cells, APCs, and anti-TCR Ab for 72 h in a classical microsuppression assay. (**B**) Mice were injected with 125,000 B16 i.d., and tumor size was measured. (**C**) Mice were injected with 125,000 B16 i.d. and sacrificed at day 12 after tumor inoculation. Microsuppression as previously described in (A) was performed. (**D**) *Foxp3*^Cre-YFP^.B6 and *Tnfsf10*^L/L^ *Foxp3*^Cre-YFP^.B6 mice were injected with 500,000 MC38 s.c. and treated with isotype or anti–PD-1 on days 6, 9, and 12 and measured for tumor growth. (**E**) CD4^+^ Foxp3^−^ and (**F**) CD8^+^ T cells were examined for percentage expression of cleaved-caspase3 (c-casp3). (**G**) CD4^+^ Foxp3^−^ and (**H**) CD8^+^ T cells were examined for percentage expression of Ki67. (**I**) CD4^+^ Foxp3^−^ and (**J**) CD8^+^ T cells from the TIL were gated for IFN-γ and TNF-α after 4-h stimulation; representative plots shown. (**K** and **L**) Tabulated data for IFN-γ and TNF-α from CD4^+^ Foxp3^−^ and CD8^+^ T cells. Data in (A) are representative of one experiment (*n* = 3–4 mice per group). Data in (B)–(L) are representative of two experiments (*n* = 6–9 mice per group). Statistics were determined using two-way ANOVA (A–D) and Student unpaired *t* test (E–H, K, and L). ns, not significant.

Our laboratory and others have suggested that T_regs_ from C57BL/6 mice can use TRAIL to suppress the immune response (42, 43). To assess this, we first examined the suppressive capacity of T_regs_ from naive *Tnfsf10*^L/L^*Foxp3*^Cre-YFP^.B6 mice. Surprisingly, the suppressive capacity of *Tnfsf10*-deficient T_regs_ was equivalent to wild-type (WT) T_regs_ (Fig. 2A). Next, to assess if T_regs_ primarily depend on TRAIL to suppress the antitumor response, we injected *Foxp3*^Cre-YFP^.B6 and *Tnfsf10*^L/L^*Foxp3*^Cre-YFP^.B6 mice with B16 melanoma. We chose this model because of studies describing the important role of T_reg_ suppression in B16 tumor growth (59, 62). However, we found no difference in B16 tumor growth in *Tnfsf10*^L/L^*Foxp3*^Cre-YFP^.B6 mice (Fig. 2B).

Furthermore, T_regs_ from the NDLN or TIL of *Tnfsf10*^L/L^*Foxp3*^Cre-YFP^.B6 mice with B16-bearing tumors were fully capable of suppressing in vitro (Fig. 2C). Moreover, the suppressive activity of T_regs_ from *Tnfsf10*^L/L^*Foxp3*^Cre-YFP^.B6 mice did not change if T_regs_ were isolated at a later time point (Supplemental Fig. 1A). We also examined an additional tumor model, MC38 colon adenocarcinoma, which has been shown to be sensitive to TRAIL-induced cytotoxicity, but found no differences in tumor growth between *Foxp3*^Cre-YFP^.B6 and *Tnfsf10*^L/L^*Foxp3*^Cre-YFP^.B6 mice (Fig. 2D) (63). In an effort to understand if T_reg_-restricted deletion of *Tnfsf10* would impact tumor growth in a model of an active immune response that justifies a strong involvement of T_reg_-mediated negative feedback, we treated *Tnfsf10*^L/L^*Foxp3*^Cre-YFP^.B6 mice with anti–PD-1 therapy and found no change in response to the immunotherapy (Fig. 2D).

T_regs_ use TRAIL to suppress through induction of cell death in CD4^+^ Foxp3^−^ T cells (42, 43). However, in *Tnfsf10*^L/L^*Foxp3*^Cre-YFP^.B6 mice, we did not find a difference in activation/cleavage of the main downstream executioner caspase 3 in CD4^+^ Foxp3^−^ or CD8^+^ T cells when compared with *Foxp3*^Cre-YFP^.B6 mice (Fig. 2E, 2F). We also assessed other immune and nonimmune populations, including tumor cells, but did not find differences in cell death (Supplemental Fig. 1B–E). This indicated that loss of TRAIL in T_regs_ did not affect cell death in immune and nonimmune populations in the TME. Interestingly, the low expression of the murine TRAIL agonistic cell DR5 may explain the lack of effect of T_reg_-mediated deletion of TRAIL (Supplemental Fig. 1F).

TRAIL can also suppress responding cells by inhibiting proliferation and T cell activation/function rather than cytotoxicity (64–67). However, the proliferation of CD4^+^ Foxp3^−^ and CD8^+^ T cells, measured by Ki67 expression, was not affected (Fig. 2G, 2H). We also analyzed the functional status of CD4^+^ Foxp3^−^ and CD8^+^ T cells and found no changes in production of proinflammatory cytokines TNF-α and IFN-γ (Fig. 2I–L). We conclude that T_reg_-restricted deletion of *Tnfsf10* does not affect T_reg_ suppression, tumor growth, cell death, or proliferation and function of T cells.

Next, we hypothesized that T_reg_-restricted deletion of TRAIL may not lead to a change in tumor growth because *Tnfsf10*^L/L^*Foxp3*^Cre-YFP^.B6 T_regs_ still retain other mechanisms of suppression. Thus, we examined the expression of suppressive molecules IL-10, LAP-TGF-β, CTLA4, CD39, and CD73, and indeed, expression was equivalent between WT T_regs_ and TRAIL-deficient T_regs_ (Supplemental Fig. 1G–K). Moreover, expression of the proliferation marker, Ki67, and markers of activation/exhaustion, PD-1 and LAG3, remained unchanged in the T_regs_ in tumors of *Tnfsf10*^L/L^ *Foxp3*^Cre-YFP^ mice (Supplemental Fig. 1L–P). These results further indicate that the suppressive phenotype of *Tnfsf10*-deficient T_regs_ is unaffected.

We also found no change in the proportion of T_regs_ or proportion of total immune cells in the tumor at day 12 (Supplemental Fig. 1Q and 1R) or day 18 (Supplemental Fig. 1S). Finally, although others have argued that TRAIL plays a role in T_reg_ apoptosis, we found no change in T_reg_ cell death in the TME (Supplemental Fig. 1T) (68). Taken together, these data suggest that T_regs_ are not primarily dependent upon TRAIL to suppress in the TME via cell death, inhibition of cell proliferation, or function. This may be due to minimal expression of DR5 and/or the use of other suppressive molecules.

### T_reg_-restricted deletion of Tnfsf10 does not affect tumor growth or suppression in BALB/c mice

**FIGURE 3.**
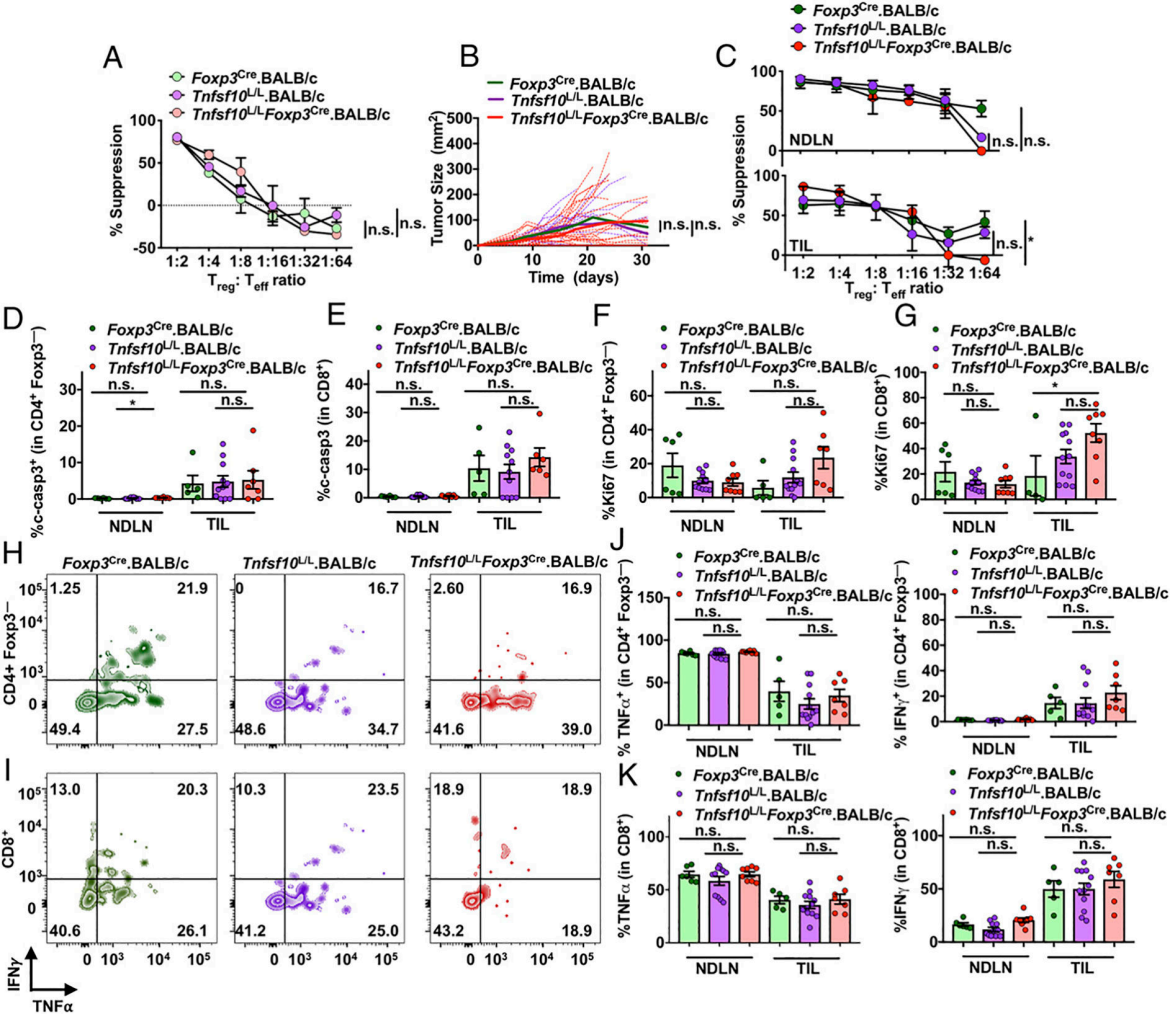
T_reg_-restricted deletion of *Tnfsf10* does not affect tumor growth or suppression in BALB/c mice. (**A**) T_regs_ (TCRβ^+^CD4^+^CD25^+^CD127^−^) were sorted from *Foxp3*^Cre^.BALB/c, Tnfsf10^L/L^.BALB/c, *Tnfsf10*^L/L^ *Foxp3*^Cre^.BALB/c naive mice and cultured with effector T cells, APCs, and anti-TCR Ab for 72 h in a classical microsuppression assay. (**B**) Mice were injected with 125,000 CT26 s.c., and tumor size was measured. (**C**) Mice were injected with 125,000 CT26 s.c. and sacrificed at day 12 after tumor inoculation. Microsuppression as previously described in (A) was performed. (**D**) CD4^+^ Foxp3^−^ and (**E**) CD8^+^ T cells from were examined for percent expression of cleaved-caspase3 (c-casp3). (**F**) CD4^+^ Foxp3^−^ and (**G**) CD8^+^ T cells were examined for percent expression of Ki67. (**H**) CD4^+^ Foxp3^−^ and (**I**) CD8^+^ T cells from the TIL were gated for IFN-γ and TNF-α after 4 h stimulation; representative plots shown. (**J** and **K**) Tabulated data for IFN-γ and TNF-α from CD4^+^ Foxp3^−^ and CD8^+^ T cells. Data in (A) are representative of one experiment with two to three mice per group. Data in (B) are representative of four experiments (*n* = 14–25 mice per group). Data in (C)–(K) are representative of two experiments (*n* = 3–12 mice per group). Statistics were determined using two-way ANOVA (A–C) and Student unpaired *t* test (D–G, J, and K). *p < 0.05. ns, not significant.

Although we did not observe a primary role for TRAIL in T_regs_ in C57BL/6 mice, we hypothesized we may see differences in BALB/c mice given our previous studies in which TRAIL had a more predominant role in BALB/c T_regs_ compared with T_regs_ from C57BL/6 mice (42). Moreover, other studies have revealed TRAIL can play a part in regulating the Th1/Th2 balance (69–72). Therefore, we backcrossed the *Tnfsf10*^L/L^ mice to the Th2-prone BALB/c background and then crossed it to the BALB/c *Foxp3*^Cre^ mouse (45). Initially, we assessed the function of naive TRAIL-deficient T_regs_ in a standard in vitro suppression assay, and interestingly, the level of suppression was equivalent to WT T_regs_ (Fig. 3A). Next, we assessed tumor growth in *Foxp3*^Cre-YFP^.BALB/c, *Tnfsf10*^L/L^.BALB/c, and *Tnfsf10*^L/L^*Foxp3*^Cre-YFP^.BALB/c mice using the BALB/c CT26 colon carcinoma model in which T_regs_ suppress the antitumor response (73, 74). Although we did not observe a difference in tumor growth (Fig. 3B), we did see a small decrease in suppression in TRAIL-deficient T_regs_ isolated from CT26 tumors compared with WT T_regs_ (Fig. 3C). However, this was not the case at a later time point (Supplemental Fig. 2A). Next, we determined that cleaved caspase levels in CD4^+^ Foxp3^−^, CD8^+^ T cells, tumor cells, and other cell populations were equivalent (Fig. 3D, 3E) (Supplemental Fig. 2B–E), suggesting that T_regs_ were not dependent upon TRAIL-mediated cytotoxicity in the TME of BALB/c mice, possibly because of low DR5 expression in the TME (Supplemental Fig. 2F).

Furthermore, we did not see any changes in Ki67, TNF-α, and IFN-γ in T cells, suggesting that T_regs_ do not suppress by limiting proliferation nor function of responding T cells (Fig. 3F–K). We also observed that TRAIL-deficient T_regs_ in the TME still retained other suppressive molecules, indicating that other molecules may aid in suppression in the TME despite loss of TRAIL (Supplemental Fig. 2G–K). Furthermore, we did not see any differences in expression of Ki67, PD-1, LAG3, and cleaved caspase 3 on T_regs_ (Supplemental Fig. 2L–Q). The proportion of immune cells and T_regs_ remained unchanged on both days 12 and 18 (Supplemental Fig. 2R–T). Taken together, these data suggest that despite the reported higher levels of TRAIL expression in BALB/c T_regs_, they are not primarily dependent upon TRAIL as a means of suppression in the TME (42).

### T_reg_-restricted deletion of Tnfsf10 does not affect autoimmune diabetes

**FIGURE 4.**
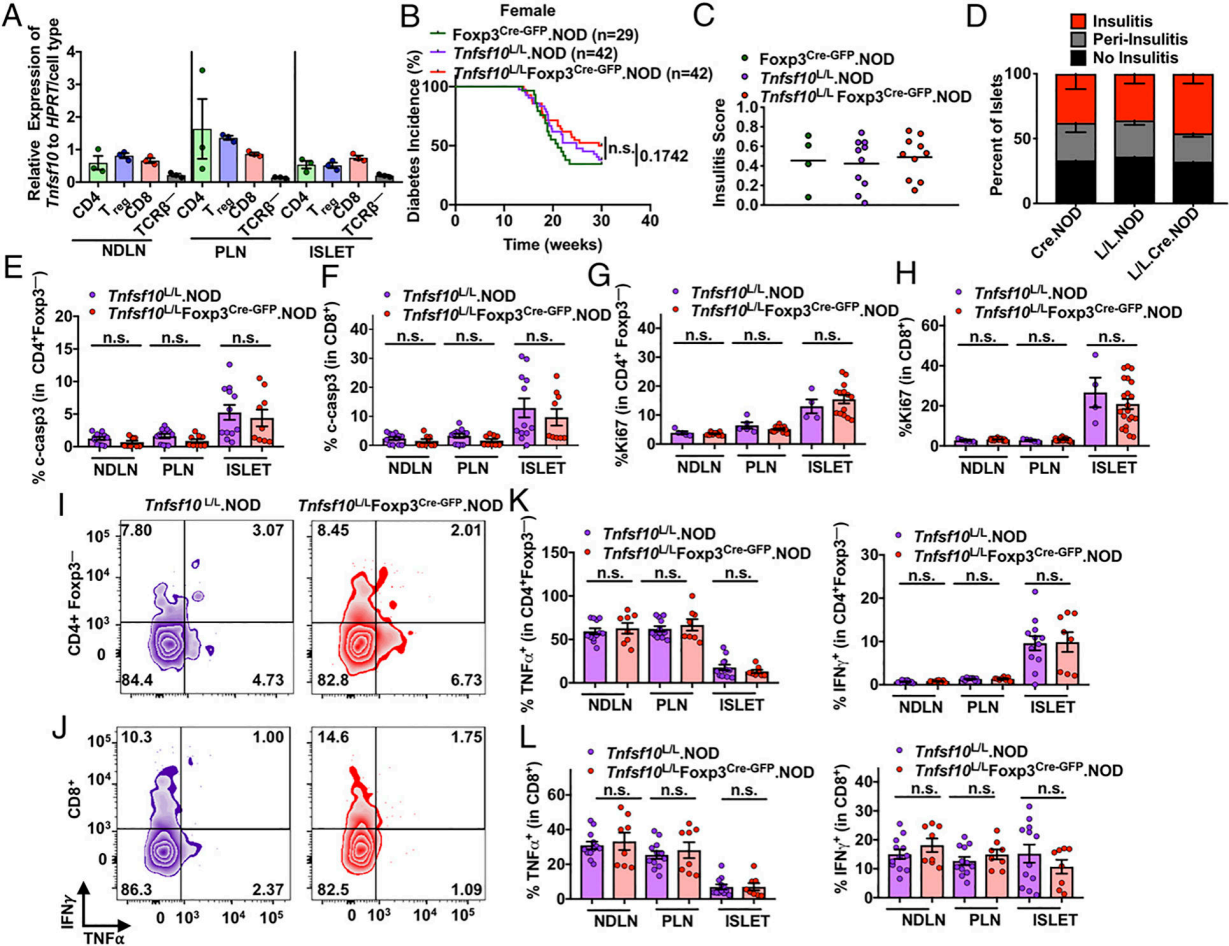
T_reg_-restricted deletion of *Tnfsf10* does not affect diabetes incidence, insulitis, or suppression in NOD mice. (**A**) Twelve-week-old female NOD Foxp*3*^Cre-GFP^ mice were sacrificed. Cells were sorted, and qPCR was performed for *Tnfsf10* and *HPRT*. (**B**) Diabetes onset monitored in *Tnfsf10*^L/L^Foxp*3*^Cre-GFP^.NOD females and cocaged controls. (**C** and **D**) Histological assessment of insulitis performed in female *Tnfsf10*^L/L^Foxp3^Cre-GFP^.NOD and cocaged controls at 12 weeks of age. (**E**) CD4^+^ Foxp3^−^ and (**F**) CD8^+^ T cells from were examined for percentage expression of cleaved-caspase3 (c-casp3). (**G**) CD4^+^ Foxp3^−^ and (**H**) CD8^+^ T cells were examined for percentage expression of Ki67. (**I**) CD4^+^ Foxp3^−^ and (**J**) CD8^+^ T cells from the TIL were gated for IFN-γ and TNF-α after 4 h stimulation; representative plots shown. (**K** and **L**) Tabulated data for IFN-γ and TNF-α from CD4^+^ Foxp3^−^ and CD8^+^ T cells. Data in (A) are representative of one experiment (*n* = 3 mice per group). Data in (B) are representative of more than three experiments (*n* = 29–42 mice per group). Data in (C) and (D) are representative of one experiment (*n* = 4–10 mice per group). Data in (E)–(L) are representative of two experiments (*n* = 4–21 mice per group). Statistics were determined using log-rank (Mantel–Cox) test (B) and Student unpaired *t* test (E–H, K, and L). ns, not significant.

Because T_regs_ are also critical in limiting autoimmunity, we hypothesized that T_regs_ may use TRAIL to suppress in the autoimmune microenvironment. Also, it has been reported that TRAIL can regulate cell death of diabetogenic T cells in the pancreatic islet of NOD mice (75). Although it was proposed that this was mediated by TRAIL-expressing pancreatic β cells, we hypothesized that T_regs_ may also use TRAIL to suppress T cells in this environment (75). Indeed, T cells express the highest levels of *Tnfsf10* in the islet (Fig. 4A). We hypothesized that T_reg_-restricted deletion of *Tnfsf10* would limit suppression of diabetogenic T cells and lead to exacerbated autoimmune diabetes.

Interestingly, we found that deletion of *Tnfsf10* in T_regs_ did not significantly alter diabetes incidence or insulitis in female (Fig. 4B–D) or male (Supplemental Fig. 3A) mice, although there was a slight trend toward reduced diabetes incidence. Moreover, we did not find any changes in cell death in CD4^+^Foxp3^−^ and CD8^+^ T cells in the islet (Fig. 4E, 4F). As seen with our tumor data, we found that the levels of proliferation and cytokine production in the diabetogenic T cells of the islet were similar in both WT and *Tnfsf10*^L/L^Foxp3^Cre-GFP^.NOD mice (Fig. 4G–L). This would indicate that T_regs_ do not require TRAIL to suppress diabetogenic T cells in the pancreatic islet of NOD mice.

We also examined DR5 expression on immune and nonimmune cells in the islet and found minimal expression of DR5 on immune cells but higher expression on insulin-positive β cells (Supplemental Fig. 3B). Reports of direct TRAIL-mediated β cell killing have been inconsistent (76–80). However, upon examination of insulin-positive cells, we found no change in cell death (Supplemental Fig. 3C). Interestingly, we did see a reduction in cell death in the CD11c^+^ population (Supplemental Fig. 3D). TRAIL can have an effect on dendritic cells (81); however, it is unclear what impact this may play in our system, as we did not see a consequence of altered disease. Future studies may elucidate what other impact this has in autoimmune diabetes.

We found that *Tnfsf10*-deficient T_regs_ isolated from the TME retained their suppressive phenotype. We questioned if this remained true for *Tnfsf10*-deficient T_regs_ isolated from the islet. We found T_regs_ still expressed functional markers such as LAP-TGF-β, IL-10, and CD39 (Supplemental Fig. 3E–G) and even had an increase in CD73 expression (Supplemental Fig. 3H). This further indicates that *Tnfsf10*-deficient T_regs_ retain their suppressive phenotype in the islet. As seen in the tumor, we found no change in T_reg_ proliferation (Supplemental Fig. 3I), as measured by Ki67, and no change in activation/exhaustion markers PD-1 and LAG3 (Supplemental Fig. 3J–M).

We had demonstrated above that TRAIL had no effect on T_reg_ cell death or the proportion of immune cells and T_regs_ in the TME. Interestingly, although we did not observe a difference in immune cell proportions within the islet (Supplemental Fig. 3N), we did see an increased proportion of intra-islet T_regs_ in *Tnfsf10*^L/L^Foxp3^Cre-GFP^.NOD mice (Supplemental Fig. 3O). Interestingly, reduced T_reg_ death was only observed in 10-wk-old mice (Supplemental Fig. 3P), as there was no difference in 12-wk-old mice (Supplemental Fig. 3Q). Therefore, we conclude that T_regs_ are not dependent on TRAIL to suppress in the islet.

Finally, we examined if T_reg_-derived TRAIL had a role in the MOG model of EAE using the *Tnfsf10*^L/L^*Foxp3*^Cre-YFP^.B6 mice. As seen with the tumor and NOD models, we did not observe a difference in EAE score and initiation of the disease between WT and *Tnfsf10*^L/L^*Foxp3*^Cre-YFP^.B6 mice (Supplemental Fig. 3R). Therefore, we conclude that T_regs_ do not require nor are dependent on TRAIL as a means of suppression in autoimmune microenvironments.

## DISCUSSION

We report four key developments from our studies. First, we created the first conditional *Tnfsf10*^L/L^ knockout mouse, that we are aware of, which allows for cell type–specific deletion of TRAIL. Although we focused our efforts on understanding TRAIL biology in T_regs_, this novel resource could be used to examine the role of TRAIL in other cell populations. Second, we used the *Tnfsf10*^L/L^ mice and determined that T_regs_ are not primarily dependent upon TRAIL as a means of suppression within the TME. Third, we found that T_regs_ from autoimmune diabetes and EAE are not primarily dependent upon TRAIL as a means of suppression. Finally, these data, along with our previous work in which multiple mechanisms of T_reg_ suppression were deleted, suggest that T_regs_ are capable of using multiple mechanisms of suppression and are able to overcome or compensate when a mechanism is compromised or blocked.

Finally, although we did not determine a primary role of TRAIL in T_regs_ within the tumor and autoimmune environments, we cannot rule out the possibility that TRAIL does play a role in T_reg_ function, either in concert with other mechanisms or in disease models we did not examine. It may be important in future studies to assess different models in which DR5 is more highly expressed. It will also be important to examine the role of TRAIL in the absence of other mechanisms of T_reg_ suppression, such as IL-10 or IL-35, in other cell types, and in other disease models such as infectious or inflammatory diseases.

## Supplementary Material

Supplement

## Abbreviations used in this article:

BMS: Bristol Myers Squibb
DR: death receptor
EAE: experimental autoimmune encephalomyelitis
hNGFR: human nerve growth factor receptor
i.d.: intradermally
MOG: myelin oligodendrocyte glycoprotein
NDLN: nondraining lymph node
PD: programmed cell death
qPCR: quantitative PCR
TIL: tumor-infiltrating lymphocyte
TME: tumor microenvironment
T_reg_: regulatory T cell
WT: wild-type
