# Supplementary material for: Regulatory T Cell–Derived TRAIL Is Not Required for Peripheral Tolerance
Source: Immunohorizons. Author manuscript; Available in PMC 2022 Jan 22. (PMC8663370; doi:10.4049/immunohorizons.2000098)
Supplement: Supplement [file NIHMS1755984-supplement-Supplement.pdf]

Supp Figure 1 Dadey et al

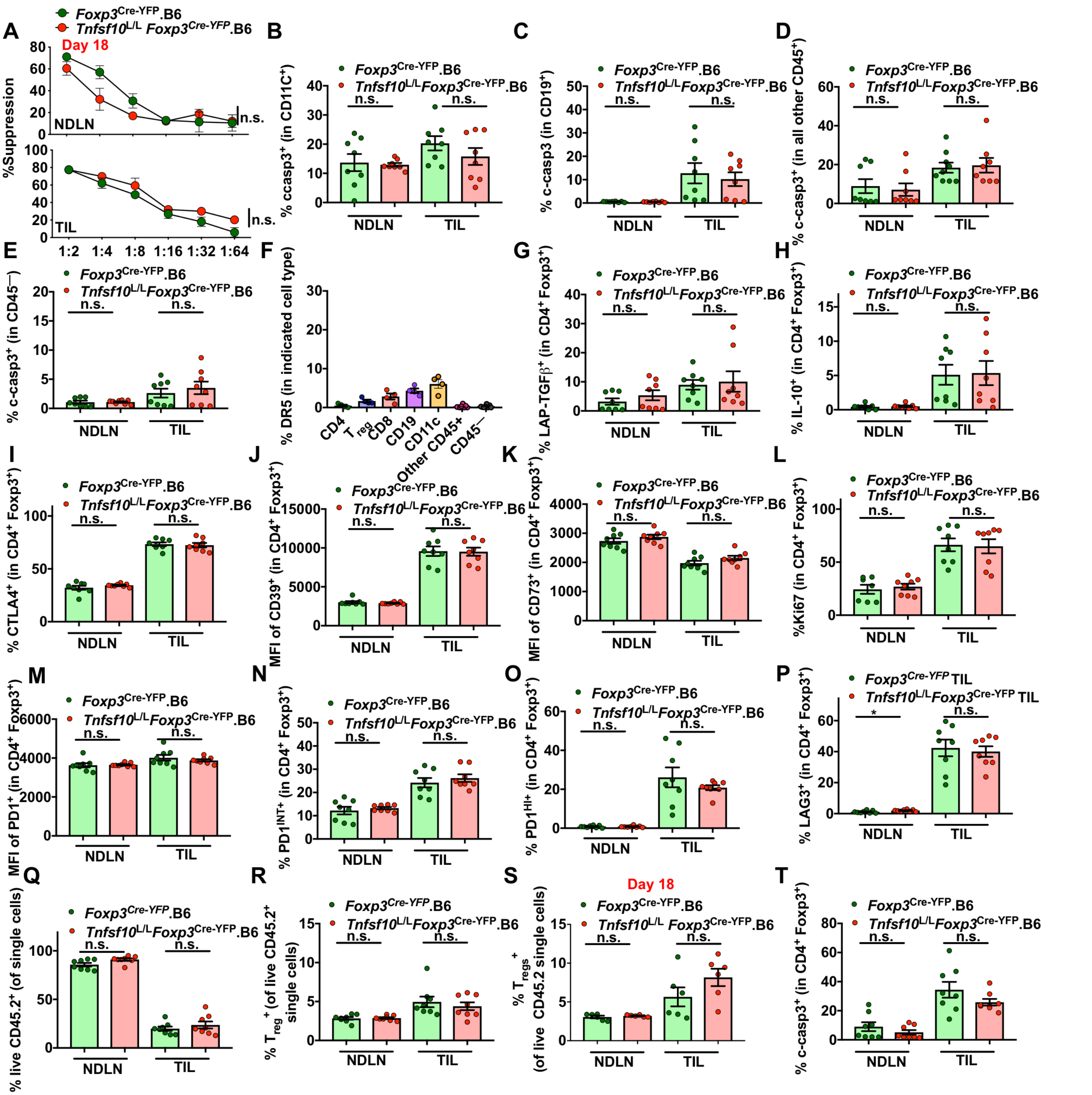

Supplemental Figure 1. No change in cell death in other populations and maintenance of other T_{reg} suppressive molecules in C57BL/6 mice
(A) *Foxp3*$^{Cre-YFP}$.B6 and *Tnfsf10*$^{L/L}$ *Foxp3*$^{Cre-YFP}$.B6 mice were injected with 125,000 B16 i.d. and T_{regs} were isolated on Day 18 from NDLN and TIL to perform a classical microsuppression assay. (B) *Foxp3*$^{Cre-YFP}$.B6 and *Tnfsf10*$^{L/L}$ *Foxp3*$^{Cre-YFP}$.B6 mice were injected with 125,000 B16 i.d. and TCRβ— CD11c^{+} cells were stained for percent expression of cleaved-caspase3 (c-casp3). (C) TCRβ—CD19^{+}, (D) CD45^{+}TCRβ—CD19—CD11c— cells and (E) CD45— cells were stained for percent expression of c-casp3. (F) Cell populations were stained for percent positive surface DR5 expression. (G) Tabulated LAP-TGFβ and (H) IL-10 percent expression on T_{regs} (I) %CTLA4 expression on gated T_{regs}. (J) Gated MFI of CD39 on T_{regs} (K) Gated MFI of CD73 on T_{regs} (L) % Ki67 on T_{regs} (M) MFI of PD-1 in PD-1^{+} T_{regs}. (N) %PD-1 intermediate in T_{regs} (O) %PD-1 high in T_{regs} (P) %LAG3 on T_{regs} (Q) % live CD45.2^{+} on gated single cells (R) %CD4^{+} Foxp3^{+} T_{regs} in live CD45.2^{+} single cells at Day 12 (S) %CD4^{+} Foxp3^{+} T_{regs} in live CD45.2^{+} single cells at Day 18 (T) %CD4^{+} Foxp3^{+} T_{regs} were gated for percent positive expression of c-casp3. Data in (A-E) (G-T) is representative of 2 experiments with 6-8 mice/group. (F) is representative of 1 experiment with 4 mice/group. 2-way ANOVA (A) was used. Student unpaired t test (B-E, G-T) was used. (ns, not significant, *p < 0.05, **p < 0.01, ***p < 0.001, ****p < 0.0001).

Supp Figure 2 Dadey et al

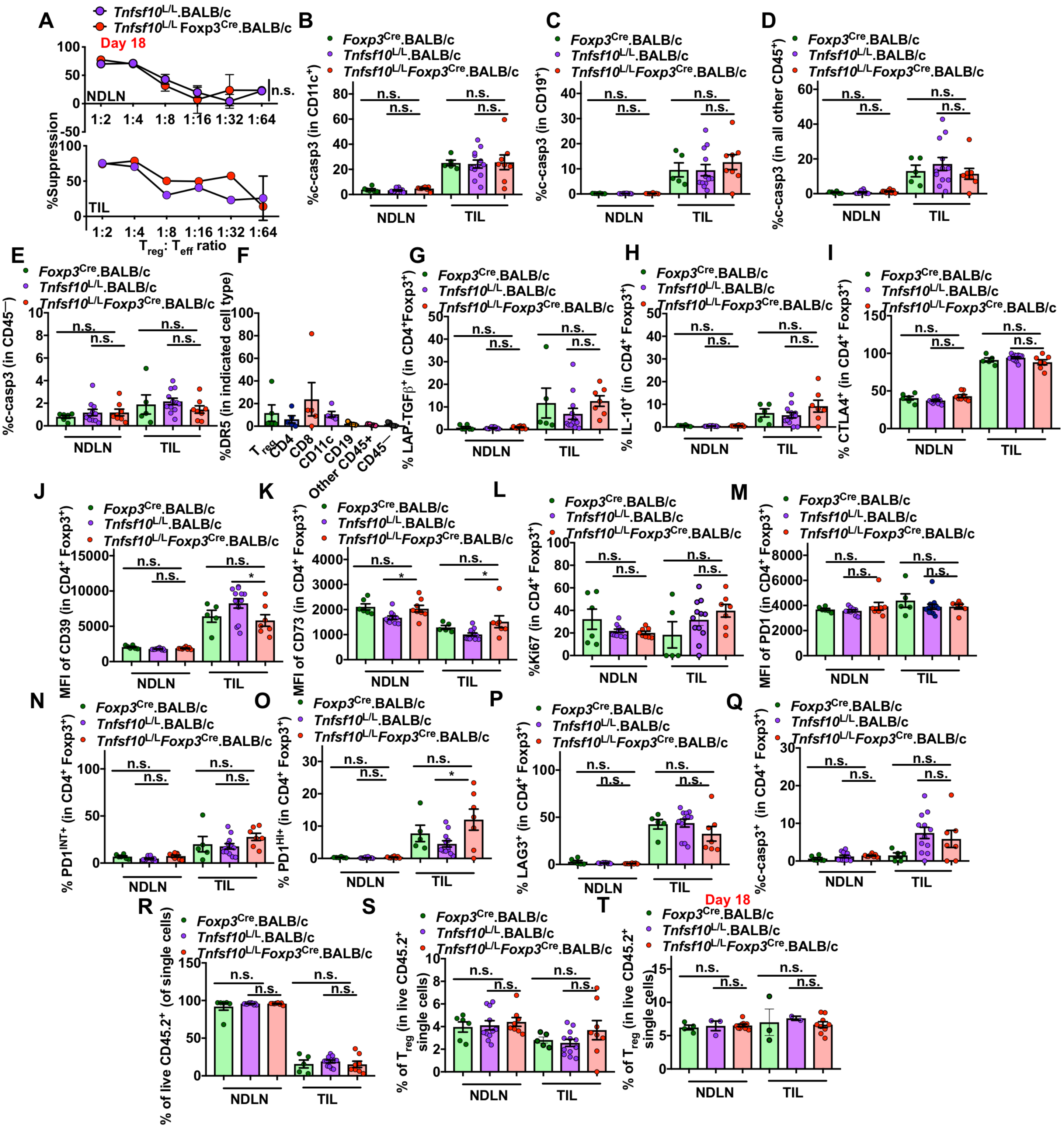

Supplemental Figure 2. No change in cell death in other populations and maintenance of other T_{reg} suppressive molecules in BALB/c mice

(A) *Tnfsf10*$^{L/L}$.BALB/c and *Tnfsf10*$^{L/L}$ *Foxp3*^{Cre}.BALB/c mice were injected with 125,000 CT26 s.c. and T_{regs} were isolated on Day 18 from NDLN and TIL to perform a classical microsuppression assay. (B) *Foxp3*^{Cre}.BALB/c, Tnfsf10$^{L/L}$.BALB/c, *Tnfsf10*$^{L/L}$ *Foxp3*^{Cre}.BALB/c mice were injected with 125,000 CT26 s.c. and TCRβ—CD11c^{+} cells were stained for percent expression of cleaved-caspase3 (c-casp3). (C) TCRβ—CD19^{+}, (D) CD45^{+}TCRβ—CD19—CD11c— cells and (E) CD45— cells were stained for percent expression of c-casp3. (F) Cell populations were stained for percent positive surface DR5 expression. (G) Tabulated LAP-TGFβ and (H) IL-10 percent expression on T_{regs} (I) %CTLA4 expression on gated T_{regs}. (J) Gated MFI of CD39 on T_{regs} (K) Gated MFI of CD73 on T_{regs}. (L) %Ki67 on T_{regs} (M) MFI of PD-1 in PD-1^{+} T_{regs} (N) %PD-1 intermediate in T_{regs} (O) %PD-1 high in T_{regs} (P) %LAG3 on T_{regs} (Q) %CD4^{+} Foxp3^{+} T_{regs} were gated for percent positive expression of c-casp3. (R) % live CD45.2^{+} on gated single cells (S) %CD4^{+} Foxp3^{+} T_{regs} in live CD45.2^{+} single cells at Day 12 (T) %CD4^{+} Foxp3^{+} T_{regs} in live CD45.2^{+} single cells at Day 18. Data in (A) is representative of 1 experiment with 2-3 mice/group pooled. (B-E, G-S) is representative of 2 experiments with 6-12 mice/group. Data in (F, T) is representative of 1 experiment with 2-9 mice/group. 2-way ANOVA (A) was used. Student unpaired t test (B-E, G-T) was used. (ns, not significant, *$p < 0.05$, **$p < 0.01$, ***$p < 0.001$, ****$p < 0.0001$).

Supp Figure 3 Dadey et al

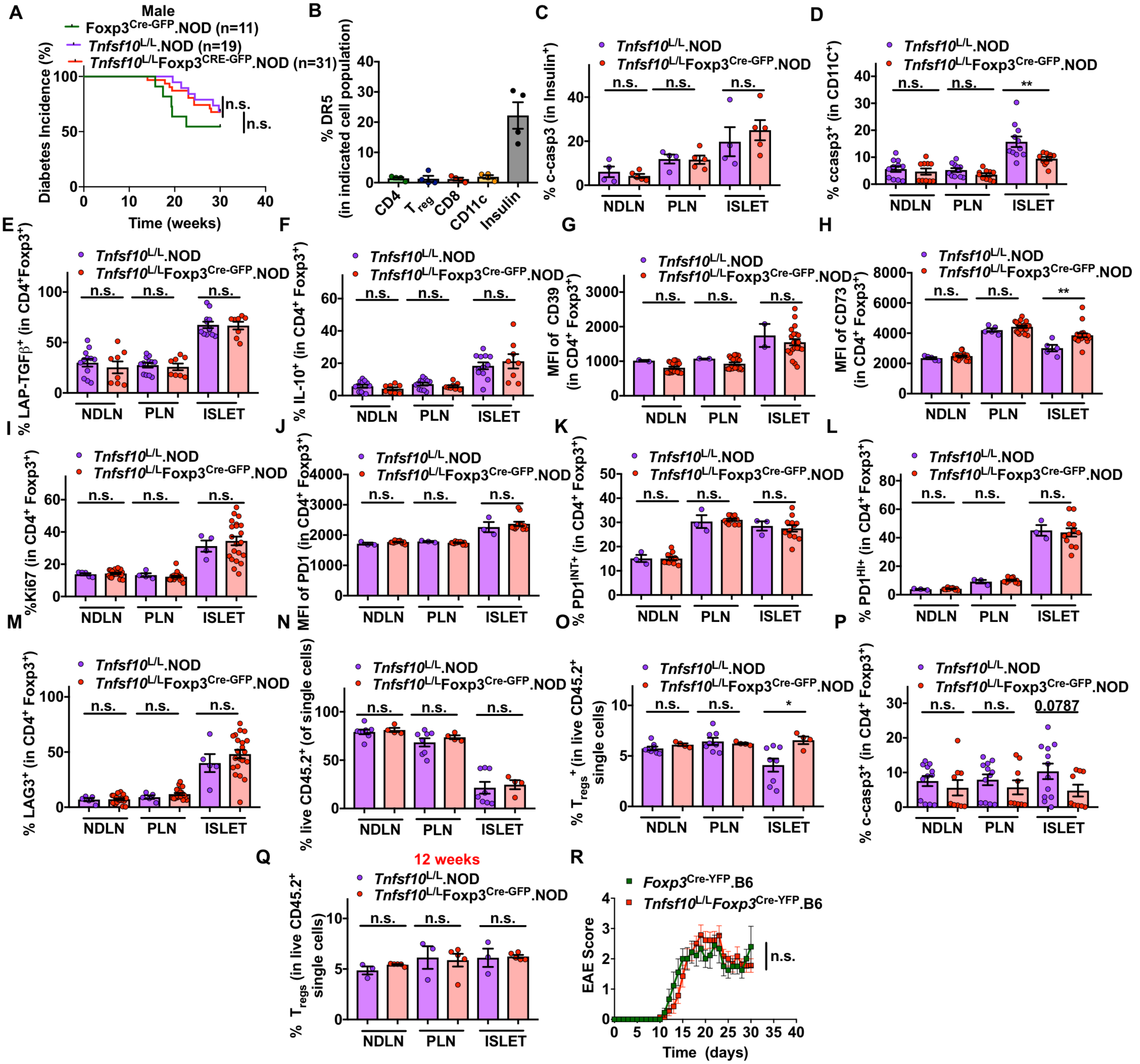

Supplemental Figure 3. T_{reg}-restricted deletion of *Tnfsf10* effects cell death of CD11c⁺ and T_{regs} in the diabetic islet
(A) Diabetes onset monitored in *Tnfsf10*$^{L/L}$Foxp3$^{Cre-GFP}$.NOD males and co-caged controls. (B) Islets from 10 week old female *Foxp3*$^{Cre-GFP}$.NOD mice were stained for surface DR5 on indicated cell populations. (C) Insulin⁺ cells isolated from *Tnfsf10*$^{L/L}$.NOD and *Tnfsf10*$^{L/L}$ *Foxp3*$^{Cre-GFP}$.NOD female mice were stained for percent expression of cleaved-caspase3 (c-casp3). (D) TCRβ⁻CD11c⁺ cells were stained for percent expression of c-casp3. (E) Tabulated LAP-TGFβ and (F) IL-10 percent expression on T_{regs} (G) Gated MFI of CD39 on T_{regs} (H) Gated MFI of CD73 on T_{regs}. (I) %Ki67 on T_{regs} (J) MFI of PD-1 in PD-1⁺ T_{regs} (K) %PD-1 intermediate in T_{regs} (L) %PD-1 high in T_{regs} (M) %LAG3 on T_{regs} (N) % live CD45.2⁺ on gated single cells (O) %CD4⁺ Foxp3⁺ T_{regs} in live CD45.2⁺ single cells at 10 weeks (P) %CD4⁺ Foxp3⁺ T_{regs} were gated for percent positive expression of c-casp3. (Q) %CD4⁺ Foxp3⁺ T_{regs} in live CD45.2⁺ single cells at 12 weeks. (R) EAE scoring in *Tnfsf10*$^{L/L}$Foxp3$^{Cre-YFP}$.B6 mice and co-caged controls.
Data in (A) is representative of >3 experiments with 11-31 mice/group. (B) is representative of 1 experiment with 4 mice/group. (C-P) is representative of 2 experiments with 4-19 mice/group. Data in (Q) is representative of 1 experiment with 3-5 mice/group. Data in (R) is representative of 3 experiments with 9-14 mice/group. Statistics were determined using Log-rank (Mantel Cox) test (A) and Student unpaired t test (C-Q) and 2-way ANOVA (R). (ns, not significant, *$p < 0.05$, **$p < 0.01$, ***$p < 0.001$, ****$p < 0.0001$).
